# Diverse Toxic Chemicals Disrupt Cell Function through a Common Path

**DOI:** 10.1371/journal.pbio.0050041

**Published:** 2007-02-06

**Authors:** Liza Gross

When technological advances in the 1930s provided the means to synthesize chemicals from petroleum and natural gas, the petrochemical industry ramped up production of diverse species of novel compounds without testing their safety. By 1940, a billion pounds of synthetic petrochemicals were produced each year. Of the more than 38 million chemicals reported in the scientific literature, 80,000 to 150,000 are used in commercial production. A report from the Harvard School of Public Health, published in The Lancet last November, warned that “a substantial number” of these chemicals—which show up in everything from cosmetics and textiles to rubber ducks and pacifiers—may be capable of damaging the human brain, particularly during development.

Any hope of developing large-scale toxicological screening methods depends on determining whether diverse toxic chemicals act through common pathways. Though few such mechanistic pathways have been identified so far, diverse toxicants can make cells more oxidized. Oxidative stress results when harmful free radicals or other “pro-oxidants” overwhelm the cell's anti-oxidant machinery, disrupting normal function. But changes in oxidative state also control multiple normal cellular functions, raising the possibility that toxicants might also affect these normal processes by making cells more oxidized. The significance of these oxidative effects—particularly, whether they might constitute a unifying principle of toxicity—has been unclear, however, in part because different toxicants increase oxidation through different mechanisms, and because the relationship between their harmful effects and their effects on oxidative status are not well understood.

In a new study, Zaibo Li and colleagues provide evidence for a novel general principle of toxicology by showing that toxicants with different chemical properties converge on activation of the same regulatory pathway with similar results. The authors monitored the response of progenitor cells isolated from the developing central nervous system to two metal toxicants, methylmercury and lead, and an organochlorine herbicide, paraquat. They found that each toxicant disrupted normal cell function by making cells more oxidized, and setting off a chain reaction that ultimately inhibited signaling pathways required for cell division. Significantly, these effects occurred at environmentally relevant, low-level exposures for lead and methylmercury.

**Figure:**
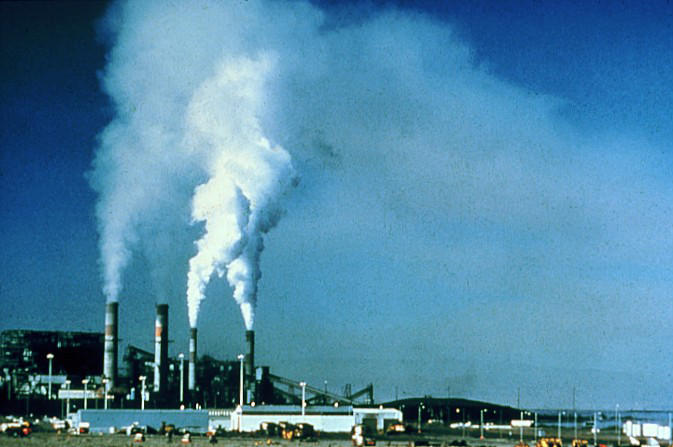
When mercury emitted from coal-fired power plants settles in wetlands or bodies of water, bacteria living in sediments transforms it into methylmercury—which is particularly toxic to the developing brain. (Photo: National Parks Service)

The authors studied the chemicals' effects in cells called oligodendrocyte precursor cells (OPCs), which give rise to the myelin-forming oligodendrocytes of the central nervous system. OPCs are particularly suited to toxicant screening, the authors explain, because of their sensitivity to small changes in oxidative (or redox) state, which determines whether the cells divide or differentiate. (When a cell or molecule undergoes an increase in oxidation state, it is oxidized; when it undergoes a decrease, it is reduced.) Previous studies by these authors have shown that redox changes in the range of 15%–20% can greatly alter responsiveness to extracellular signaling molecules, and that such changes may help regulate the normal development of these cells. For example, OPCs that are slightly more reduced undergo extensive cell division when grown in the presence of the cell-division stimulator platelet-derived growth factor (PDGF). In contrast, OPCs that are more oxidized, within normal physiological redox ranges, undergo differentiation.

Exposing OPCs to low levels of methylmercury, similar to those found in the environment, made the cells 20% more oxidized—and inhibited cell division (as indicated by the number of cells in the synthesis phase of the cell cycle). For example, methylmercury levels as low as 20 nM (equivalent to four parts per billion) caused a 25% drop in the percentage of cells that underwent cell division in response to PDGF stimulation. Each year, methylmercury is detected in the cord blood of 600,000 infants at levels equal to or above those producing these effects.

Reduced cell division, the authors show, resulted from disrupted PDGF signaling: proteins normally activated by PDGF signaling (for example, Erk1/2 and Akt) were inhibited by exposure to methylmercury (analyzed at the still sublethal exposure level of 30 nM), which also reduced the abundance of PDGF's receptor, PDGFRα. To find out which part of the PDGF pathway methylmercury targeted, the authors exposed OPCs to neurotrophin-3 (NT-3), a signaling molecule that also activates Erk1/2, a downstream component of multiple signaling pathways. Since neither Erk1/2 nor TrkC, the NT-3 receptor, were affected by the toxicant, they concluded that it must act upstream of this component.

These results suggested that methylmercury might activate an enzyme—the ubiquitin ligase c-Cbl—that targets PDGFRα for degradation. This possibility seemed especially attractive since c-Cbl doesn't target TrkC and is activated by Fyn, an enzyme previously shown to be activated by oxidative stress. And that's what the authors found: methylmercury activates Fyn, which then activates c-Cbl, which in turn reduces PDGFRα levels by targeting the receptor for degradation. Methylmercury's effects could be blocked by suppressing Fyn or c-Cbl with appropriate molecular constructs, by overexpressing PDGFRα, or by inhibiting Fyn activation with the pharmacological blocker PP1.

The pro-oxidants lead and paraquat (which differ chemically both from each other and from methylmercury) produced the same effects as methylmercury on OPCs and on the PDGF pathway (about 20% increased oxidation, Fyn and c-Cbl activation, Erk1/2 suppression, and reduced PDGFRα levels). Fyn activation, and all its observed consequences, could be blocked by neutralizing the redox changes caused by all three toxicants with the anti-oxidant drug N-acetyl-L-cysteine. Additional experiments showed that the toxicants caused similar reductions in two other targets of c-Cbl (c-Met and EGFR), further bolstering the hypothesis that these diverse toxicants converge on the same pathway to disrupt cell function.

But could these results predict the effects of toxicant exposure in developing animals? To find out, the authors exposed mice to methylmercury from conception to 21 days after birth (by providing the chemical to mothers in their drinking water). As predicted by the OPC experiments, the mice had reduced levels of the two c-Cbl targets, PDGFRα. and EGFR, in the tissue of three brain regions. In contrast, TrkC levels, which c-Cbl does not target, were unaffected—just as predicted by the in vitro experiments. Methylmercury produced these effects, and also reduced OPC division in vivo, at doses 75%–90% below those previously considered a low dose for mice.

Sixty years after the dawn of the petrochemical era, health effects are known for just a fraction of its bounty. And many of these chemicals—including lead, mercury, and a long list of organochlorines—now even contaminate human breast milk. While this study demonstrates that even low levels of diverse toxicants can disrupt the developing nervous system, it also provides a framework for analyzing a wide range of toxic chemicals based on their pro-oxidant activity. In so doing, it provides a strategy for quickly assessing the physiological effects of many of the toxic chemicals pervading our environment—the first step in identifying agents that might protect the most vulnerable among us.

